# The causal relationship between obstructive sleep apnea and otitis media: a bidirectional Mendelian randomization study

**DOI:** 10.3389/ebm.2025.10540

**Published:** 2025-11-13

**Authors:** Ruixin Guo, Yifan Zhang, Yijie Chen, Wenqi Sha, Wanyi Kou, Chensi Xu, Yuran Lei, Ningrui Zhang, Liu Yang, Yun Guo, Huihui Zhang, Zhenghui Wang

**Affiliations:** 1 Department of Otolaryngology-Head and Neck Surgery, The Second Affiliated Hospital of Xi’an Jiaotong University, Xi’an, China; 2 Shaanxi Provincial Key Laboratory for Precision Diagnosis and Treatment of Otorhinolaryngology, The Second Affiliated Hospital of Xi’an Jiaotong University, Xi’an, China

**Keywords:** obstructive sleep apnea, otitis media, mendelian randomization, sleep disorder, twosample mendelian randomization

## Abstract

Obstructive sleep apnea (OSA) is manifested as periodic collapse of the upper airway during sleep. Otitis media is a spectrum of infectious and inflammatory diseases involving the middle ear. In this study, we sought to determine the causal effect of OSA on otitis media using a bidirectional, two-sample Mendelian randomization (MR) analysis. We analyzed the data from two different, extensive genome-wide association studies (GWAS) and selected OSA-related single-nucleotide polymorphisms (SNPs) as instrumental variables (IVs). Bidirectional MR analysis was conducted using the inverse-variance weighted (IVW) method. To ensure the robustness of the results, alternative sensitivity analysis procedures were performed, including MR-Egger, the MR pleiotropy residual sum and outlier (MR-PRESSO), and leave-one-out analysis. In the forward MR analysis, OSA was correlated with an increased risk of acute suppurative otitis media (odds ratio, 1.164; 95% confidence interval, 1.056–1.283; *P* = 0.002) and suppurative and unspecified otitis media (odds ratio, 1.150; 95% confidence interval, 1.059–1.249; *P <* 0.001). All reverse MR analyses showed that otitis media had no causal effect on OSA (*P* > 0.05). The MR analysis supports that OSA contributes to the development of otitis media. Thus, managing OSA may be beneficial in treating otitis media.

## Graphical Abstract

**Figure:**
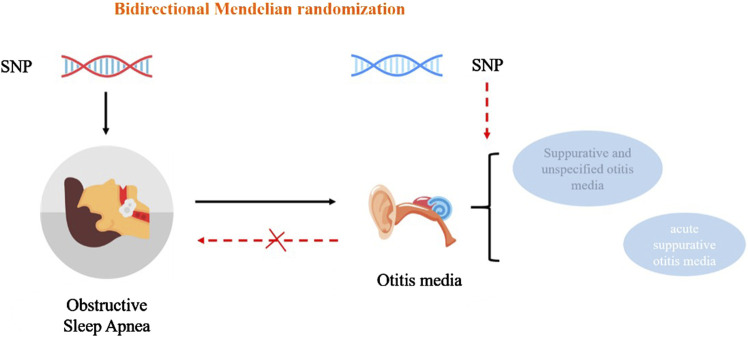

## Impact statement

Several other studies have linked OSA to the development of otitis media, and similar pathological changes occur during the pathogenesis of both OSA and otitis media, such as the presence of systemic inflammation. Accordingly, we speculate that there may be a connection between OSA and otitis media, which confirmed by the result of the bidirectional MR analysis. Our finding may be exploited to improve the detection and management of otitis media.

## Introduction

Obstructive sleep apnea (OSA) is a common type manifested as periodic collapse of the upper airway during sleep [1]. The sleep disorder is related to intermittent hypoxia, which elicits systemic inflammatory responses by promoting the release of inflammatory mediators such as nuclear factor-κB (NF-κB), interleukin (IL)-6, and IL-1β [2]. Accordingly, OSA may lead to serious complications affecting the cardiovascular, endocrine, and neurological systems [3–5].

Otitis media is a spectrum of infectious and inflammatory diseases involving the middle ear [6]. Risk factors for otitis media include bacterial or pathogenic infections, allergies, nasal congestion (sinusitis, adenoid hypertrophy, nasal or nasopharyngeal tumors), ciliary dysfunction, and possibly gastroesophageal reflux disease (GERD) [7]. Otitis media can cause a variety of pathological consequences, such as meningitis, acute mastoiditis, and hearing loss. Identification of the causal factors for otitis media is essential to improve the prognosis of patients with otitis media [7].

The relationship between OSA and otitis media is still controversial. On the one hand, from a macro-disease perspective, a previous study suggests no link between OSA and otitis media [8]. However, several other studies have linked OSA to the development of otitis media [9, 10]. OSA-related adenoid hypertrophy contributes to eustachian tube dysfunction, thus resulting in the development of secretory otitis media, particularly in children [9, 10]. Similar pathological changes occur during the pathogenesis of both OSA and otitis media, such as the presence of systemic inflammation [7, 11] and endoplasmic reticulum stress [12]. On the other hand, genetics play a role in both OSA and otitis media. Research had revealed that families with a history of chronic and recurrent otitis media have a higher incidence of otitis media than the general population [13]. A GWAS on OM was conducted at the University of Pittsburgh (UPitt), and significant duplication of rs10497394 on chromosome 2 was shown in a population of OM families. It is thought that this SNP plays a role in regulation by altering the binding of transcription factors, epigenetic markers, or lamellipodia-associated structural domains [14]. Similarly, OSA is a genetically complex disease that may result from the interaction of multiple genetic and environmental factors [15]. The role of genetic factors in OSA susceptibility is also supported by the studies of familial aggregation [16]. In addition, inflammation-related SNPs are associated with otitis media and OSA. Studies have shown that the risk of OM may be increased by SNPs for the IL-6 (−174) and proinflammatory cytokines tumor necrosis factor (TNF) [17]. It was also shown that polymorphisms in the TNF-α gene were associated with obstructive sleep apnoea (OSA) [18]. Therefore, we hypothesized that OSA may be associated with the development of otitis media. However, there is still a lack of appropriate clinical studies to explore this causal relationship further.

Mendelian randomisation (MR) analysis is an approach to investigate the causal link between illness exposure and outcome using an instrumental variable (IV) - genetic variation [3]. MR is based on the idea that genetic variation is randomly distributed to offspring and has an advantage over traditional observational research in reducing confounding effects and reverse causation [19]. In this study, we analyzed the data from two large genome-wide association studies (GWAS) and chose OSA-related single-nucleotide polymorphisms (SNPs) as IVs. We aimed to determine the causal relationship between OSA and otitis media using a bidirectional MR analysis. Understanding the risk factors associated with otitis media can be beneficial for early diagnosis and treatment.

## Materials and methods

### MR research design

**FIGURE 1:**
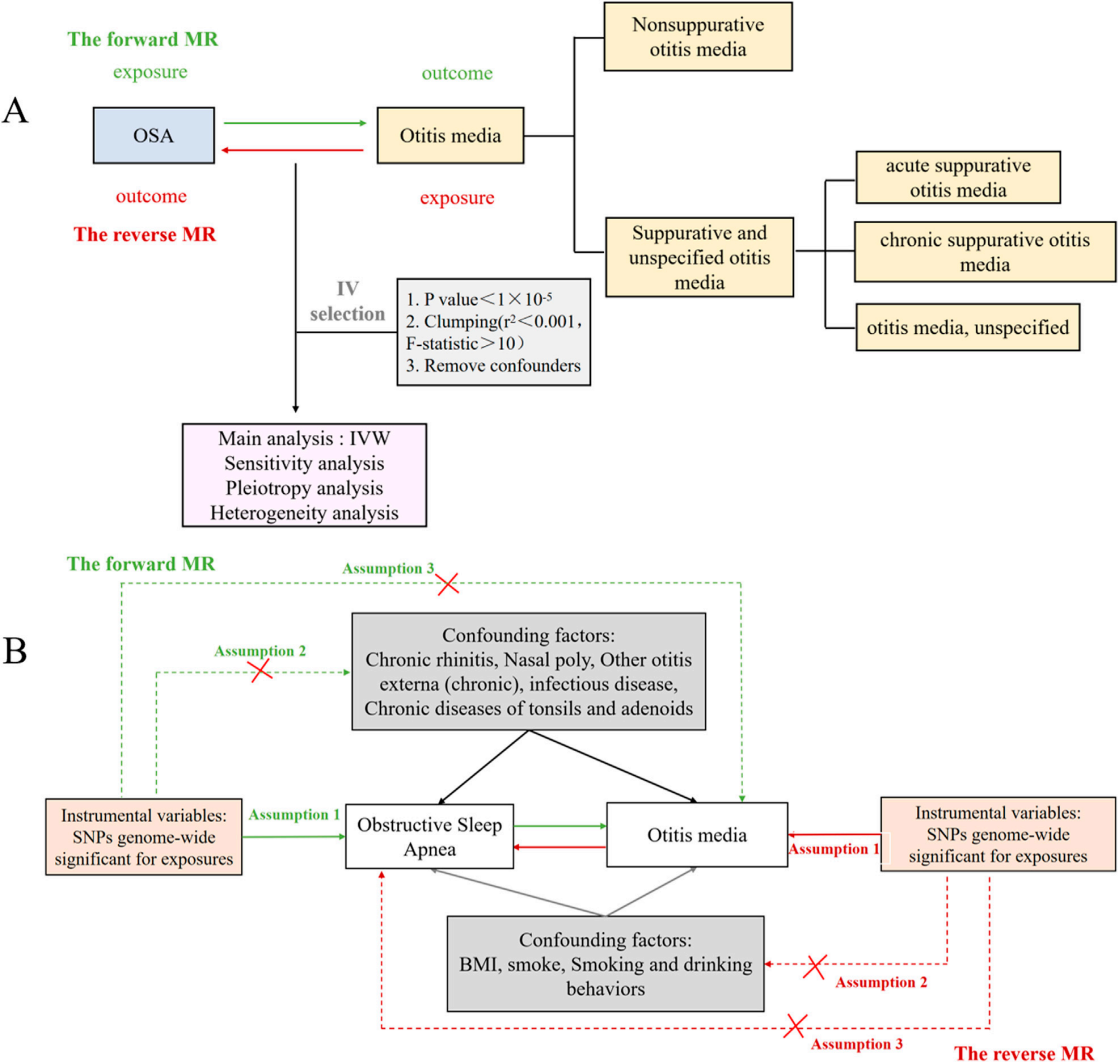
The Mendelian randomization study aims and assumptions. **(A)** The Mendelian randomization study aims. **(B)** The Mendelian randomization study assumptions.

To determine the causal relationship between OSA and otitis media, we performed a bidirectional MR analysis (Figure 1A). SNPs were separately selected as IVs for the datasets of OSA and otitis media with their subtypes, while confounders were removed based on a review of the literature. There are three hypotheses for the MR analysis: (1) IVs are strongly associated with exposure; (2) IVs are not associated with confounders that may affect exposure or outcome; (3) IVs affect otitis media only through the exposed pathway rather than other pathways (Figure 1B).

### Data sources

We extracted the clinical and laboratory data on OSA and otitis media from FinnGen Study^1^ and GWASs databases. This data was collected when all participants gave informed consent in their original studies. There is no requirement for additional ethical approval since reliance is only on summary-level statistics. Considering that bias can be caused in the estimates by population mixing, the genetic background of the population in the MR study was restricted to those of European descent.

#### Genetic summary-level data of OSA

Genetic predictors of OSA were obtained from FinnGen Study (G6_SLEEPAPNO). This dataset was built by the Finnish National Gene Research Project and contained 217,955 Europeans (16761 cases and 201,194 controls) with 16,380,465 SNPs. The case group comprised 10,557 males and 6,204 females. The average age at the first event was 54.91 years for males and 56 years for females. The overall unadjusted incidence rate was 7.72%, with 11.21% and 5.05% for males and females, respectively. The population diagnosis for the case group was based on ICD codes, all of which were ICD-10: G473. According to the American Academy of Sleep Medicine guidelines, diagnostic testing for OSA should be performed in conjunction with a comprehensive sleep evaluation and adequate follow-up, and polysomnography is the standard diagnostic test for the diagnosis of OSA in adult patients in whom there is a concern for OSA based on a comprehensive sleep evaluation [20]. The apnea-hypopnea index (AHI) is the primary measure of OSA severity, as indicated by the PSG outcomes [1]. AHI of ≥5 events/h with symptoms of respiratory sleep disorders and associated conditions, or AHI of ≥15 events/h without associated symptoms or conditions, fits the diagnostic criteria for OSA [21].

#### Genetic summary-level data of otitis media

The classification of otitis media into suppurative and non-suppurative (ICD-10) has been standardized by the International Health Organization (IHO), and further classification of otitis media into acute and chronic according to the course of the disease and the type of exudate has been carried out. In 2002, Gates et al. made further adjustments to the classification standard [22]. They proposed that otitis media should be divided into acute and chronic according to the course of the disease. They also suggested that otogenic complications should be categorized. This classification standard is now widely used.

However, based on the Mendelian randomization analysis that we performed using data from the FinnGen Study^1^ 和IEU openGWAS of GWAS database^2^, the classification and diagnostic criteria for otitis media were dominated by the ICD-10. The ICD-10 staging criteria include Nonsuppurative otitis media and Suppurative and unspecified otitis media. The latter includes acute suppurative otitis media, chronic suppurative otitis media and otitis media, unspecified.

First, we chose the datasets of Suppurative and unspecified otitis media (H8_MED_SUPP). The datasets contained 213,184 Europeans (7245 cases and 205,939 controls) with 16,380,441 SNPs. The first subtype dataset of Suppurative and unspecified otitis media was acute suppurative otitis media (finn-b-H8_SUP_ACUTE), which contained 211,171 Europeans (5,232 cases and 205,939 controls) with 16,380,429 SNPs. The case group comprised 2328 males and 2904 females. The average age at the first event was 14.55 years for males and 16.94 years for females. The overall unadjusted incidence rate was 2.41%, with rates of 2.47% and 2.36% for males and females, respectively. The population diagnosis for the case group was based on ICD codes, all of which were ICD-10: H660, ICD-8: 3810. The next subtype was Chronic suppurative otitis media, which was obtained from a publicly available GWAS dataset (GWAS ID: ebi-a-GCST90018809) and statistically analyzed by Sakaue S (PMID: 34594039) [23], including 484,145 Europeans (1,108 cases and 483,037 controls) and 24,194,289 SNPs. The last subtype was otitis media, unspecified, and its source was the FinnGen Study (H8_OTIMEDNAS). The Finnish National Gene Research Project built this dataset and comprises 2,179,555 European individuals (1,832 cases and 205,939 controls), with a total of 16,380,419 SNPs. The case group comprised 745 males and 1,087 females. The average age at the first event was 24.24 years for males and 24.48 years for females. The overall unadjusted incidence rate was 0.84%. Rates for males and females were 0.79% and 0.88%, respectively. The population diagnosis for the case group was based on ICD codes, all of which were ICD-10: H669, ICD-8: 3819.

According to ICD-10 classification criteria, the next subtype of otitis media was nonsuppurative otitis media (H8_NONSUPPNAS). The dataset of nonsuppurative otitis media contained 210320 Europeans (4,381 cases and 205,939 controls) with 16,380,433 SNPs. The case group comprised 1854 males and 2527 females. The average age at the first event was 21.78 years for males and 21.19 years for females. The overall unadjusted incidence rate was 2.02%, with rates of 1.97% and 2.06% for males and females, respectively. The population diagnosis for the case group was based on ICD codes, all of which were ICD-10: H65, ICD-9: 381.

### IV selection

In our study, we initially used a stringent threshold of *P* < 5 × 10^−8^ to screen for SNPs that were strongly associated with OSA and Otitis media, but only a few SNPs could fit this criterion. Finally, we set the significance threshold to *P* < 1 × 10^−5^ in OSA-otitis media studies and Otitis media-OSA studies. To avoid bias caused by linkage disequilibrium, any SNP that met the significance requirement must also have a r^2^ value <0.001 and a kb value >10,000. We then removed palindromic SNPs with moderate allele frequencies, significant outliers detected by the MR Pleiotropy REsidual Sum and Outlier (MR-PRESSO) analysis, and confounders indicated by the Ldlink tool^3^. F values were calculated using formula F = (β/SE) [2], where β is the SNP’s impact value and SE is its standard deviation [24]. The weak IVs with the F value of <10 were excluded [25].

### MR analysis

In this study, we used the R software (version 4.3.3), the TwoSampleMR software package (version 0.5.10) for MR analysis, and the MR-PRESSO software package (version 1.0) for outlier removal. The inverse variance weighted (IVW) approach was utilized as the primary analysis method, with the MR-Egger, weighted median, simple model, and weighted model serving as auxiliary methods.

### Sensitivity analysis

We employed the IVW and MR-Egger methods to assess heterogeneity. The heterogeneity among IVs was investigated using Cochran’s Q statistic. We utilized a random-effects model in the MR analysis to eliminate heterogeneity-related bias. The test threshold for heterogeneity was *P >* 0.05. The MR-Egger intercept and MR-PRESSO were used to assess pleiotropy. MR-Egger is an adaptation of Egger’s regression model which considers horizontal pleiotropy by incorporating an intercept into the weighted regression model [26]. The MR-PRESSO method was used to improve the analysis by identifying and excluding outliers that could be due to pleiotropy. The MR-PRESSO outlier test required at least 50% of the variants to be valid instruments dependent on Instrumental Strength Independent of Direct Effects (InSIDE), meaning the effect size of a variant on exposure should not depend on a horizontal, multidirectional effect on the outcome [27]. Funnel plots were used to ensure the study results were consistent and reliable. A “leave-one-out” analysis was also implemented to test the robustness and consistency of the results. The MR results for the remaining instrumental variables were calculated by excluding individual SNPs on a one-by-one basis, in order to assess whether the SNPs influenced the association between OSA and the risk of otitis media. The test threshold for pleiotropy was *P >* 0.05.

## Results

### IV selection for MR analysis

In the OSA-otitis media investigation, 59 SNPs were obtained after chain imbalance screening, and 10 were excluded due to their association with otitis media. Finally, we chose 49 SNPs as the IV, with an F-value of >10 for each SNP. The SNPs selected are given in Supplementary Table S1. In the otitis media - OSA investigation, we utilized a similar strategy for SNP selection. The final SNPs used for MR analysis are provided in Supplementary Table S2.

### Causal effects of OSA on otitis media

**FIGURE 2:**
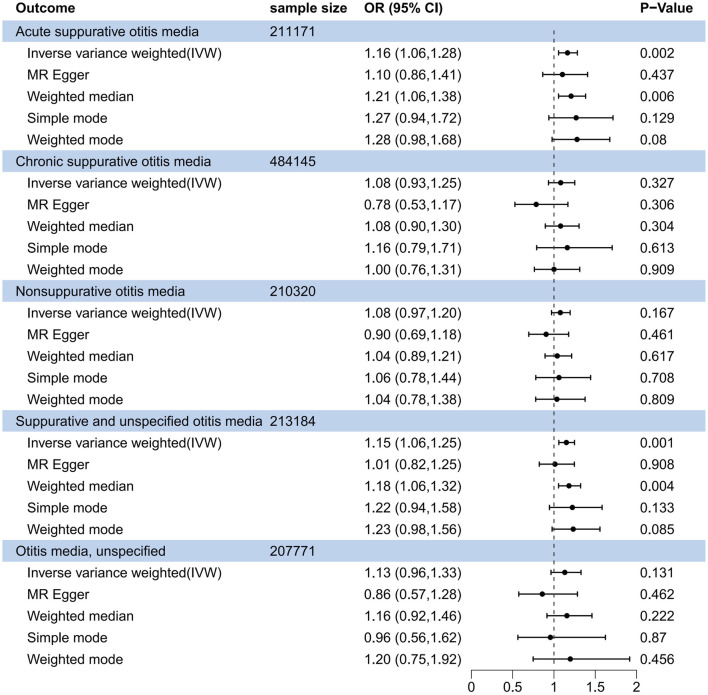
Forest plot of causal association between obstructive sleep apnea and suppurative otitis media in the forward MR analysis.

In the forward MR analysis (Figure 2), the IVW method indicated OSA as a risk factor for acute suppurative otitis media (odds ratio (OR), 1.164; 95% confidence interval (CI), 1.056–1.283; *P* = 0.002). The Weighted median method also revealed a significant causal relationship (OR, 1.208; 95% CI, 1.056–1.715; *P* = 0.006). The association of OSA with suppurative and unspecified otitis media was also uncovered by the IVW (OR, 1.150; 95% CI, 1.059–1.249; *P* < 0.001) and Weighted median (OR, 1.182; 95% CI, 1.056–1.249; *P* = 0.004) methods. Both Weighted median IVW methods displayed similar scatter plots and had no significant heterogeneity or pleiotropy (*P* > 0.05; Supplementary Figure S1). Leave-one-out sensitivity analysis revealed that removing any single SNP had no significant effect on the results (Supplementary Figure S2). In addition, the other three subtypes of otitis media, i.e., chronic suppurative otitis media, nonsuppurative otitis media, and unspecified otitis media, did not appear to be related to OSA.

### Causal effects of suppurative otitis media on OSA

**FIGURE 3:**
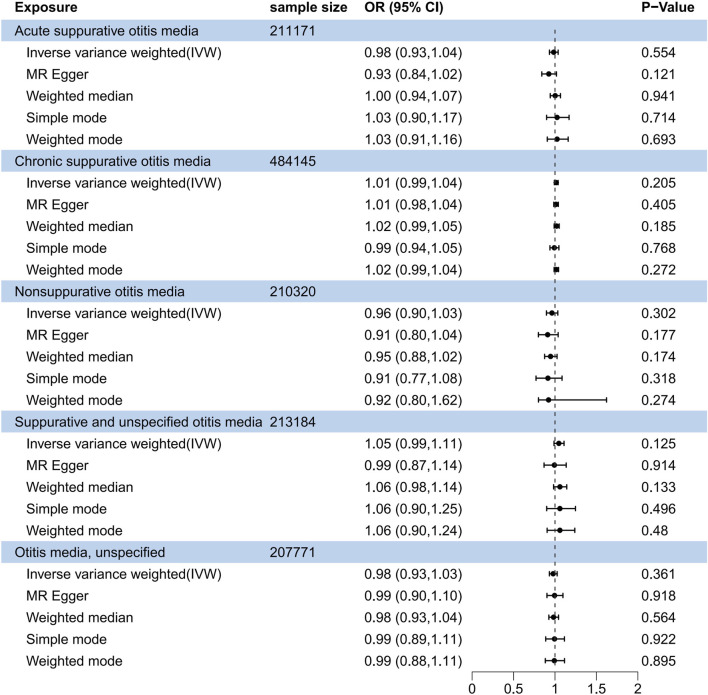
Forest plot of causal association between obstructive sleep apnea and suppurative otitis media in the reverse MR analysis.

In the reverse MR analysis, none of the suppurative otitis media subtypes showed a causal relationship with OSA (Figure 3). The OR for the acute suppurative otitis media - OSA relationship was 0.984 (95% CI, 0.993–1.038; *P* = 0.554) in the IVW method, 1.015 (95% CI, 0.992–1.038; *P* = 0.205) for chronic suppurative media - OSA, 0.963 (95% CI, 0.897–1.034; *P* = 0.302) for nonsuppurative otitis media - OSA, 1.046 (95% CI, 0.988–1.109; *P* = 0.125) for suppurative and unspecified otitis media - OSA, and 0.978 (95% CI, 0.932–1.026; *P* = 0.361) for otitis media, unspecified - OSA. Similar results were obtained when the Weighted median method was used.

## Discussion

In this work, we performed the MR analysis based on a large GWAS dataset to evaluate the causal relationship between OSA and otitis media. Our findings support the idea that OSA is significantly related to the development of otitis media, but otitis media has no causal effect on OSA.

Although our results show that the threshold for selecting SNPs is 1 × 10^5^, the F statistics of the selected SNPs are all >10. Furthermore, the F statistics of the final 49 SNPs from positive Mendelian randomization after removing the confounders are all >19. This indicates that all our selected SNPs are strong instrumental variables, which provides a solid basis for conducting correlation analyses. Then, SNPs that were strongly associated with otitis media, including chronic rhinitis, nasal polyps, other otitis externa (chronic), infectious disease, and chronic diseases of tonsils and adenoids, were selected to avoid the influence of confounding factors. The final results show that the selected SNPs can only cause otitis media through OSA, and the forward MR results indicate that OSA can cause otitis media. Our results suggest that OSA is a risk factor for otitis media. This may be explained by the involvement of inflammatory and/or infectious processes in the pathogenesis of both OSA and otitis media. Abnormalities in nasopharyngeal anatomy contribute to the development of OSA. It has been documented that children with AOM had a reduced nasopharyngeal height and a small nose angle [28]. Newborns with OSA present an increased incidence of pharyngeal and eustachian tube dysfunction [29]. The angle between the anterior and medial skull bases, the depth of the maxilla, and the height of the upper face are associated with the development of otitis media in adults [30]. Functional defects in the nasopharynx play an important role in the development of suppurative otitis media, which may arise from aberrant opening of the eustachian tube and bacterial colonization of the nasopharynx [31]. OSA is associated with pressure changes in the upper respiratory tract. The Eustachian tube not only protects the middle ear from bacterial infection, but also regulates pressure balance in the middle ear cavity [7]. OSA-induced pressure change in the upper respiratory tract may lead to the dysfunction of the Eustachian tube, consequently facilitating the development of suppurative otitis media [32].

In addition, our study shows no significant relationship between OSA and persistent suppurative otitis media. Recurrent middle ear infection has been suggested to contribute to chronic suppurative otitis media [33]. It has been documented that patients with chronic otitis media have a higher abundance of *Haemophilus* influenzae than patients with OSA [34]. Thus, rather than OSA, chronic suppurative otitis media may be caused by bacterial infection.

There are some limitations in this study. Firstly, our analysis was performed on only the European population. On the one hand, the prevalence of obstructive sleep apnoea (OSA) and otitis media varies across populations. Studies have shown that the prevalence of OSA is highest in China, followed by the United States, Brazil, and India [35]. A review of the literature has shown that the prevalence of OSA in Asian adults ranges from 3% to 97% [36]. In contrast, the prevalence of OSA in the general adult population in Europe ranges from 9% to 38% [37]. Significant differences in these results may be related to differences in geography, ethnicity, and research design. As for otitis media, it has also been shown that in Asia-Pacific countries, the prevalence of OM in schoolchildren ranges between 3.25% (Thailand) and 12.23% (Philippines) [38]; Auinger et al. showed that the prevalence of OM in children younger than 6 years of age in the United States was 68.2% (95% CI: 66.3%, 70.1%) during the period 1988–1994 [39]. Furthermore, there are discrepancies in the pathogens that cause acute otitis media. Research conducted in the United States, Finland and the Netherlands has demonstrated that in children between 4 weeks and 18 years of age, the predominant pathogens of AOM are *Streptococcus* pneumoniae (23%–48%) and *Haemophilus* influenzae (41%–57%) [40]; nevertheless, one study has shown that the predominant pathogens of AOM in Chinese children under 18 years of age are *Streptococcus* pneumoniae (47.2%; 108/229) and *Staphylococcus aureus* (18.8%; 43/229) [41]. These imply that the progression of OSA and otitis media is connected to a variety of elements, among which ethnic variations play a significant role. On the other hand, studies in a single population are insufficient to reveal all disease variants in different populations. GWA studies have been successful in identifying genetic variants that contribute to complex human traits, but they have mainly focused on European populations. The results of GWA studies may be affected by differences between populations, such as variations in disease allele frequencies, linkage disequilibrium (LD) patterns, phenotypic prevalence, effect sizes and rare variants [42]. To achieve a more comprehensive understanding of human genetic variation, it is essential to expand GWA studies to include more non-European populations. Secondly, the study subjects were adults. Since otitis media is more common in children, a validation study should be performed in Children. It is also necessary to include a wider range of people, including individuals of various age groups, in the GWA studies.

## Conclusion

Our bidirectional MR analysis reveals a causal link between OSA and otitis media. This finding may be exploited to improve the detection and management of otitis media. The particular mechanism underlying the relationship between OSA and otitis media deserves further investigation.

## Data Availability

The original contributions presented in the study are included in the article/Supplementary Material, further inquiries can be directed to the corresponding author.

## Footnotes

1
https://www.finngen.fi/en

2
https://gwas.mrcieu.ac.uk/

3
https://ldlink.nih.gov/

## Supplementary material

SUPPLEMENTARY FIGURE S1 The scatter plots of OSA on suppurative otitis media (Acute suppurative otitis media and suppurative and unspecified otitis media).

SUPPLEMENTARY FIGURE S2 The Leave-one-out sensitivity analysis plots of OSA on suppurative otitis media (Acute suppurative otitis media and suppurative and unspecified otitis media).

SUPPLEMENTARY TABLE S1 The final snp of OSA database.

SUPPLEMENTARY TABLE S2 The final snp of five subtype of otitis media.
