# Supplementary figures and images for: The causal relationship between obstructive sleep apnea and otitis media: a bidirectional Mendelian randomization study
Source: Exp Biol Med (Maywood). 2025 Nov 13;250:10540. doi: 10.3389/ebm.2025.10540 (PMC12658985; doi:10.3389/ebm.2025.10540)

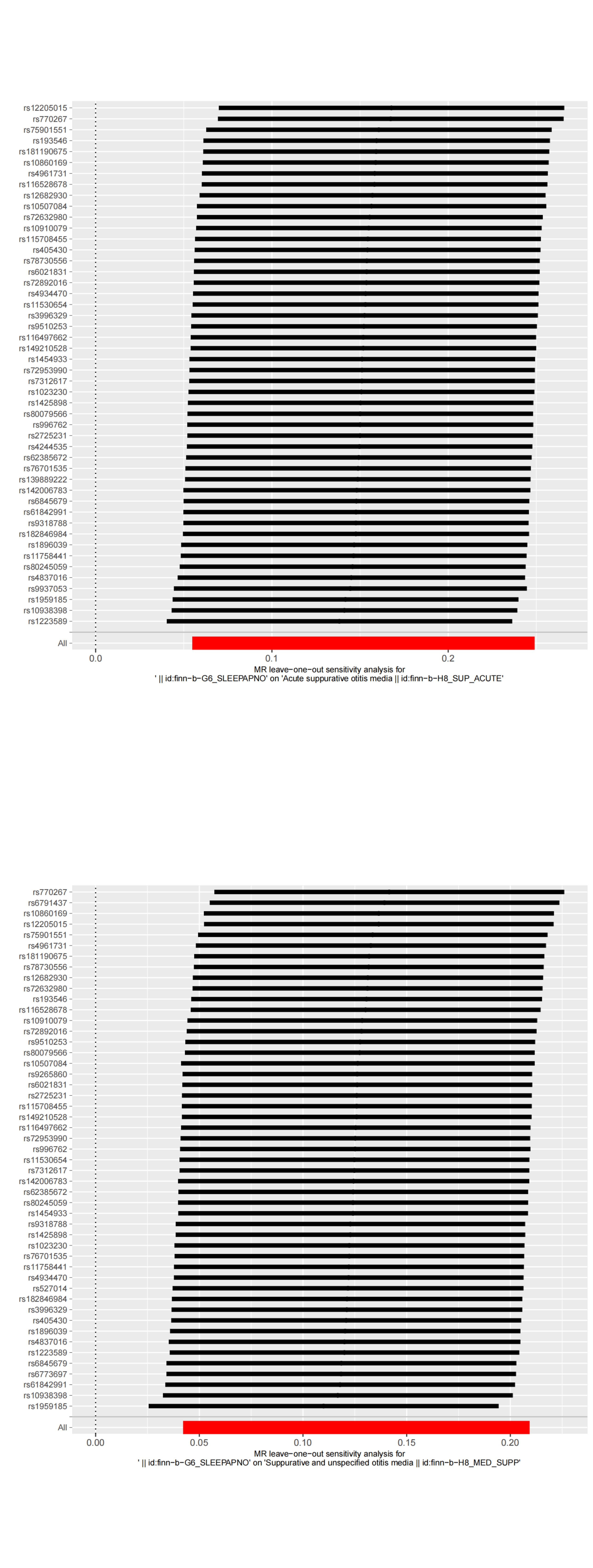

Supplement: Supplementary file 2 [file Image2.jpg]

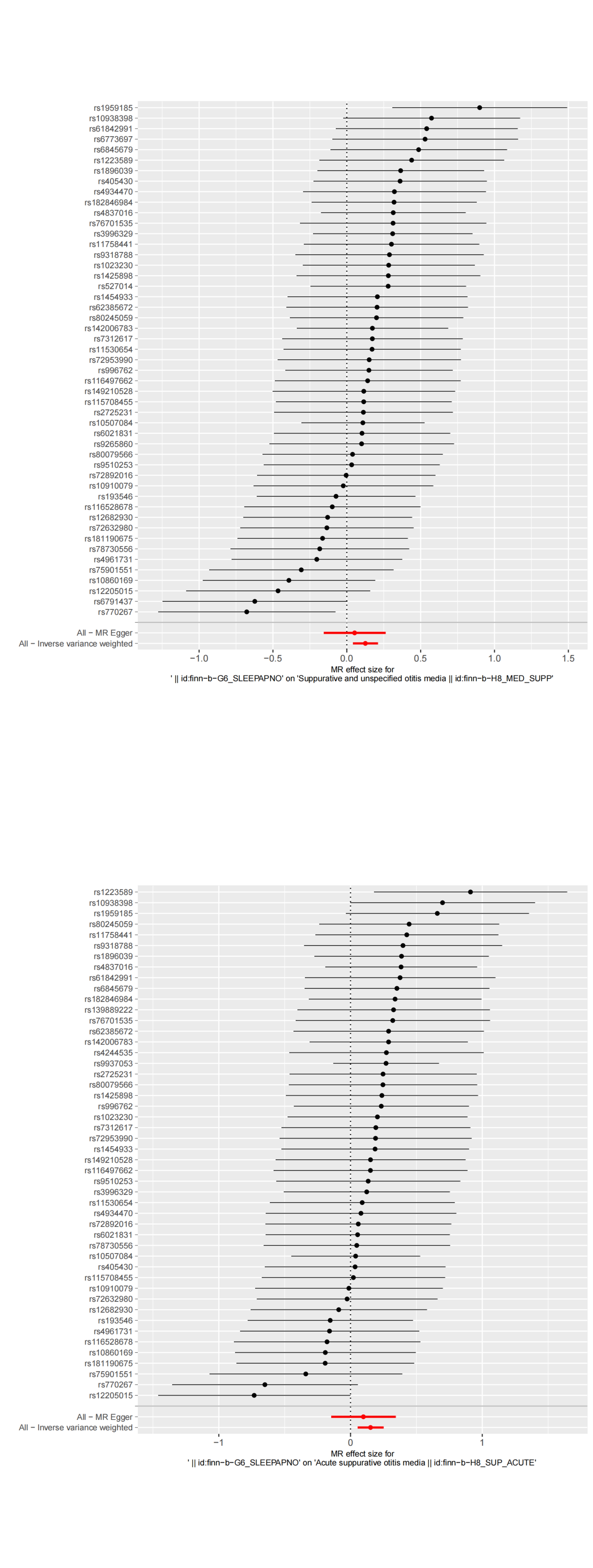

Supplement: Supplementary file 4 [file Image1.jpg]
